# An efficient modular framework for automatic LIONC classification of MedIMG using unified medical language

**DOI:** 10.3389/fpubh.2022.926229

**Published:** 2022-08-10

**Authors:** Surbhi Bhatia, Mohammed Alojail, Sudhakar Sengan, Pankaj Dadheech

**Affiliations:** ^1^Department of Information Systems, College of Computer Science and Information Technology, King Faisal University, Al Hasa, Saudi Arabia; ^2^Department of Computer Science and Engineering, PSN College of Engineering and Technology, Tirunelveli, India; ^3^Department of Computer Science and Engineering, Swami Keshvanand Institute of Technology, Management & Gramothan (SKIT), Jaipur, India

**Keywords:** MedIMG, deep learning, LIONC, accuracy, natural language processing

## Abstract

Handwritten prescriptions and radiological reports: doctors use handwritten prescriptions and radiological reports to give drugs to patients who have illnesses, injuries, or other problems. Clinical text data, like physician prescription visuals and radiology reports, should be labelled with specific information such as disease type, features, and anatomical location for more effective use. The semantic annotation of vast collections of biological and biomedical texts, like scientific papers, medical reports, and general practitioner observations, has lately been examined by doctors and scientists. By identifying and disambiguating references to biomedical concepts in texts, medical semantics annotators could generate such annotations automatically. For Medical Images (MedIMG), we provide a methodology for learning an effective holistic representation (handwritten word pictures as well as radiology reports). Deep Learning (DL) methods have recently gained much interest for their capacity to achieve expert-level accuracy in automated MedIMG analysis. We discovered that tasks requiring significant responsive fields are ideal for downscaled input images that are qualitatively verified by examining functional, responsive areas and class activating maps for training models. This article focuses on the following contributions: (a) Information Extraction from Narrative MedImages, (b) Automatic categorisation on image resolution with an impact on MedIMG, and (c) Hybrid Model to Predictions of Named Entity Recognition utilising RNN + LSTM + GRM that perform admirably in every trainee for every input purpose. At the same time, supplying understandable scale weight implies that such multi-scale structures are also crucial for extracting information from high-resolution MedIMG. A portion of the reports (30%) are manually evaluated by trained physicians, while the rest were automatically categorised using deep supervised training models based on attention mechanisms and supplied with test reports. MetaMapLite proved recall and precision, but also an F1-score equivalent for primary biomedicine text search techniques and medical text examination on many databases of MedIMG. In addition to implementing as well as getting the requirements for MedIMG, the article explores the quality of medical data by using DL techniques for reaching large-scale labelled clinical data and also the significance of their real-time efforts in the biomedical study that have played an instrumental role in its extramural diffusion and global appeal.

## Introduction

The digitization of documents has created plenty of new opportunities for liberalising data collections for NLP pipelines like machine translation as well as web search engines. Automatically creating captions for a certain image is problematic, while the foundation of understanding the scene is partly due to computer vision's exceptional achievement. Books, manuscripts, letters, bills, catalogues, and other digital documents (MedIMG) are machine-printed, handwritten, or radiology images (1). Learning effective representations of document images that are rich in preserving both lexical as well as semantic information is the only way to gain access to content-level in such a vast numerical corpus. In this paper, we focus on encoding the word MedIMG in a featuring space that conserves its verbal data, and we limit our stability by only using the number of declarations. The restricted availability of large-scale labelled datasets within the medical realm justifies their construction, as they play a vital role in developing medicine using Machine Learning (ML) techniques (2). Clinical text data like physician notes as well as radiology reports must be annotated using labels that include specific information, namely the kind, features, and anatomical area of disease, for more effective use. However, since text contains highly specialised language, ambiguous terms, and narrative sentences, labelling text is challenging and must be done manually by health experts. Some of the things that make it hard for clinical text to be used in considerable medical research and mostly with DL approaches is that it does not have annotations (3).

This study can be presented as a completely innovative descriptor of word pictures that may be utilised to construct MedIMG-based applications (X-rays, handwritten and printed documents, radiology images) (4). To obtain the UMLS concept, annotations/captions use picture data, which will subsequently be compared to the images' original text captions. The images utilised are clinically relevant radiological images, and the captions were written by medical professionals. Moreover, DL approaches are used to extract medical knowledge using clinical documents. We believe that the classification of scanned documents into words is provided to us either through underlying data or through an external word suggestion approach, which may be chaotic. In this paper, a word-level description is presented as a model. It has to do with extracting information from medical records that include radiographs. In addition, MetaMapLite pays attention to real-time processor speed and then begins with a restricted range of essential functionalities like the longest-term match as well as negation detection. MetaMapLite is presented in this work, and its execution equals that of the existing Prologue operation of MetaMap is assessed (5). We examine whether employing high-resolution MedIMG rather than downscaled and low-resolution MedIMG can lead to improved clinical work performance. We anticipate that increasing the size of an image would improve MedIMG segmentation's work performance (6). Throughout all input resolutions, the experimental results show how to stack groups across determinations beat each discrete learner. We additionally give easily understandable Ensemble Model (EM) scale weights, implying that multi-scale features are required for automated detection of a higher level of MedIMG. Extensive research is being conducted in the field of handwritten character recognition. Handwritten character recognition has been developed by many users. A few systems have been examined: Using fuzzy logic, a character recognition system was created (7). They have designed a system that is compatible with VLSI technology. Their system for recognising characters can't be changed or messed up. They have incorporated hammering NN into their system. It has been found that NN can be used to recognise handwritten characters in a novel way. A Kohonen Self-Organising Map (SOM), an unsupervised NN, has been utilised.

The following contributions are focused on this paper:

- Information Extraction from Narrative Clinical Texts: An automatically generated mapping system connects basic phrases as well as codes collected throughout several clinical terminology and programming systems for Systematised Terminology of Medicine-Clinical Terminology concepts to standardise multi-site heterogeneous Electronic Healthcare Record (HER).
- Automatic Classification for MedIMG with Image Resolution: Next, an innovative large-scale dataset of X-rays is used to accurately assess its chances of attaining increased clinical work performance using high-resolution visuals rather than downscaled low-resolution MedIMG. MedIMG, as well as findings of multi-label annotations, are being developed.
- Hybrid Model to Predictions of Named Entity Recognition (NER)by RNN + LSTM + GRM: Our goal is to present an innovative UMLS-based RNN+LSTM+GRM for NER tool and compare its outcome to state-of-the-art, broadly utilised, accessible public medical NER tools.

Apart from trying to implement and obtain results for clinical testing and MedIMG, this article refers to the study of health data, the innovation of using DL methods for obtaining large-scale labelled MedIMG datasets (8), as well as the relevance of its applications in the medical research that has played a role towards its extramural diffusion and global reach. This paper summarises the journey in such a way that the reader is guided through multiple disciplines, from engineering to medicine, all the way up to the ethical implications of implementing Artificial Intelligence (AI) in treatment.

## Literature review

The health care industry must have commonly recognised and advertised the partial use of EHR systems throughout the previous decade. The Health Information Technology to Economic and Clinical Health (HITECH) Act of 2009 (9) motivated medical hospitals and clinics to implement EHR systems. Though HER systems are planned for operational purposes, they were later used to process data. All of the information recorded by the EHR system could now be analysed. The enormous data acquired by progress notes, combined with the rapid adaptation of EHR systems, has resulted in a significant research area of predictive medical analytics, which employs narrative progress notes. For many years, conventional Machine Learning (ML) models were used to perform predictive analysis, mainly in the medical domain. Because of the operational excellence of DL methods in recent times, many have been widely used in biomedical disease prediction. For example, using EHR data, researchers created a Long Short-Term Memory (LSTM) network model for predicting heart failure. A Deep Convolutional Neural Network (CNN) is used to predict diabetes. In addition, a CNN methodology to detect colorectal cancer, which uses diagnoses as well as medication of patients in the HER, was developed (9). Using real-world Pashto/Urdu text, another research was performed, and this text can be rotated in any direction, not just in a straight line. The results were analysed using various ML techniques, including Scale Invariant Feature Transformation (SIFT), LSTM, and Hidden Markov Model (HMM) to detect rotated text. In this study, the LSTM got 98.9% accuracy, while HMM-based techniques got 89.9% accuracy and SIFT got 94.3% accuracy.

Multiple successful efforts were made in the earlier days, focusing on various factors like data modality (radiology and handwritten images), nature of depiction (firmed/adjustable length), and also by categorising of entrenching structure. It is divided the debate of associated studies are: (a) traditional techniques developed by means of flexible length depiction plans, (b) static length depiction attained by means of a bag of words structure, and (c) different classifier models with learned representation implemented on a maximum of hand-crafted features as well as in DL networks (10).

A data analysis Language Model (LM's) goal is to approximate the probabilistic model on classifications of distinct entities or tokens. This work includes a variety of Natural Language Processing tasks (NLP), and the majority of NLP tasks necessitate the use of NLP models. An NLP model shown in such a task describes the possibility distribution function of word sequences and stemmed word-token sequences (11). This approach's efficacy was tested using a variety of datasets, including three well-known Handwritten Text Recognition (HTR) datasets, as well as three cutting-edge Optical Models for text recognition and eight different spellcheck correction techniques, including both traditional statistics and current approaches utilising NN in NLP. They made a model for spelling correction that was tested statistically with HTR system metrics. In the tests, the average sentence correction rate was 54% higher than with the most advanced decoding method. An LM, on the other hand, can define probability distribution function on sequences for some other type of token, like bytes/characters. So, this is the issue of LM character-level, which models language to be distribution functions on sequences to characters rather than words- for example (12), without a clear and specific notion of sentence-level boundaries. It has been demonstrated that for learning to forecast the next character based on earlier characters, such models also can understand inner depictions, which obtain syntactic and semantic properties and then produce accurate grammar transcripts with words, subclauses, quotations, and sentences.

Word Embeddings (WE) (13), recognised as word vectors and distributed representations, are complex depictions of words in a low-dimensional actual vector space that overcome the dimensionality blow of n-grams. Neural Word Embeddings are those WE studied by a Neural Network (NN). In terms of computer visualisation, these embeddings are analogous to image embeddings acquired as images in CNN models' hidden layers.

Convolutional neural network has been used to process the data with a grid-like structure and spatial patterns. CNNs have been effectively applied to various forms of data wherever they can be presented as discrete units grouped in periodic intervals together in a grid-like variety of dimensions. The local spatial features of images are determined by CNN and reported to generate relatively high features. The first CNN layer generates feature maps that include spatial features that were extracted locally. This shows how high-level features and local spatial features are made. Despite the fact that they were initially developed for picture data, it is presented as a 2-D set of pixels. Data of Time-series, which is presented like the 1-D grid with examples collected during periodic intervals, then text, which can be thought of like a 1-D grid of ordered tokens, are two examples (13).

Recurrent Neural Networks (RNN) are developed to evaluate sequential data by recursively sharing the same weights across successive time steps “t.” At each time step, a member of an input sequence is therefore fed into the network. RNNs can be designed in various ways based on recurrent connexions within their units. LSTM (14) is a sort of gated RNN which uses self-loops to create routes with long gradient flows and has been found to learn long-term dependency easier than vanilla RNN designs. Gated RNNs provide temporal pathways with a derivative that does not explode or vanish, similar to leaky units. These pathways include connexion weights that can vary for every time step, enabling knowledge to be accumulated or forgotten by erasing the previous state.

A Multi-Label Text Classification (MLTC) (15) task is one that involves extracting many medical categories from radiological data. MLTC is indeed an NLP task that allows you to apply one or even more labels per document. This differs significantly from the typical binary but rather multi-class classification issues that have been extensively researched inside this machine learning literature (16). Binary classifier treats classes as separate target variables, which is naturally inefficient for MLTC because label dependencies cannot be exploited. A mutually exclusive assumption concerning classes is used by multi-class classifiers. According to this concept, each document should have one class, which is not valid in multi-label setups. This section's related works are primarily concerned with MLTC (16). A novel method for recognising handwritten characters has been advanced using NN. They used an unsupervised NN called the Kohonen Self-Organising Map (SOM). They developed a system that can be used for character recognition as well as the recognition of other Indic languages. If the handwritten characters are not effectively segmented, the system occasionally provides false results. One of the authors can use the handwriting of a human to verify their authenticity. In their system, the author has implemented an FFNN with multiple layers. One sophisticated hypothesis in this paper is that the handwritten alphabet's height and width are unique to the writer's individual hand size and handwriting. It has been demonstrated that a patient's handwriting can be used to identify them. Hyper-parameters and model parameters can differ significantly, as demonstrated by (7). Some hyper-parameters, such as the number of epochs, hidden units, hidden layers, Learning Rate (LR), kernel size, and activation function, must be set before training begins. It is stated that if the hyper-parameters are selected improperly, CNN performance may suffer. Some CNN models have 27, 57, 78, and 150 hyper-parameters, respectively, for AlexNet, VGG-16, GoogleNet, and ResNet-52. In the field of handwriting recognition, implementing researchers play a big role in making sure that CNN parameters are set up well so that recognition performance can be improved.

We present an automated picture denoising method based on quasi-periodic denoising. The Fourier transformation is used to detect the image's high amplitude noisy spectrum. The proposed model for noise filtration has a threshold value of 30. The threshold value is updated in response to user input. Thresholding, as well as filtration-based models, are used to minimise the noisy components (17). Finally, an adaptable restoration filter is used to obtain the noise profile's exact outline. A method for denoising images using a quicker and more flexible CNN is provided. This study takes into account various levels of noise that use a non-uniform noise level map to eliminate the spatial noise. For applying local low-priority matrix recovery with global spatial-spectral variation, a hyperspectral image denoising algorithm is suggested. A unified mixed Gaussian noise plus sparse noise removal model is recommended in this research article. To separate the filtered image regions from sparse noise, the hyperspectral images are first segmented into overlapping local patches, and thus the matrix recovery scheme is used. This work rebuilds the image with global acceptance (18). As a result of this, a machine is trained to correctly identify Pashto handwritten characters that have never been seen before. With the help of this dataset (7), we trained and tested three FFNN models (Model 1 with a 1-ReLU layer, Model 2 with 2-ReLU layers, and Model 3 with 3-ReLU layers) using the backpropagation algorithm. Sample images of user-defined handwritten text were used to test the proposed overall system performance. This preprocesses images to prepare them for CNN training (19). The input document is then segmented by line, word, and character. Multiple experiments yielded results deserving of high recognition. At one point in the character segmentation method, they were able to get up to 86% accuracy.

The labelled datasets of the most frequent named entity recognition tasks are used widely and are publicly available. CoNLL-03, a shared study from the Conference on Computational NLP Learning in 2003, the MUC-7 dataset (20), and also the Onto Notes corpus consists of around ten named entity types as well as seven various entity forms like TIME and DATE, which are the three most common corpora used to train such extractors. The jobs are typically modelled using BIO upgraded labels–separate labels for the beginning, inside, and outside tokens of an entity.

## Mathematical model

### Generation of MedIMG caption

#### Encoder of convolutional features

Our model provides a caption X that is encrypted as a series of 1-n encrypted words from a single input image Eq. (1).


(1) $$\begin{array}{r} X=\left\{X_{1},...X_{C}\right\},X_{i}\equiv R^{n} \end{array}$$

WHERE the size of the syntax by K and the length of the tagline by C.

To retrieve a sequence of a feature vector that refers to an annotation vector (21), we employ a CNN. The extractor generates Y (vectors); each one is the d-dimensional description of image segmentation, Eq. (2)


(2) $$\begin{array}{r} Y=\left\{Y_{1},...Y_{L}\right\},Y_{i}\equiv R^{d} \end{array}$$

In contrast to prior work, which used a fully connected layer, we extract the features from such a lower convolutional layer to derive a correspondence between feature vectors and sections of the 2-D image. By weighting a subclass of whole feature vectors, the interpreter can preferentially focus on some areas of a picture.

#### Decoder of LSTM

We apply an LSTM network to construct captions by producing one word at a time based on the context vector, the initial Hidden State (HS) (22), and earlier generated comments, Eq. (3)


$$\begin{array}{rcl} a_{t} & = & \left(P_{i}Q_{t-1}+R_{t}T_{t-1}+S_{i}s_{t}+b_{i}\right)\\ b_{t} & = & \left(P_{i}Q_{t-1}+R_{t}T_{t-1}+S_{i}s_{t}+b_{f}\right) \end{array}$$

(3) $$\begin{array}{rcl} c_{t} & = & f_{t}c_{t-1}+i_{t}+\tanh\left(P_{c}Q_{t-1}+R_{c}S_{t-1}\right) \end{array}$$

$$\begin{array}{rcl} d_{t} & = & \left(P_{i}Q_{t-1}+R_{t-1}+b_{i}\right)\\ e_{t} & = & O_{t}+\tanh\left(c_{t}\right) \end{array}$$

The input, output, forget, HS, and memory of the LSTM are represented by at, bt, ct, dt, and et, respectively. P∙, Q∙, R∙, and S∙ are weight matrices as well as biases that have been learned. An embedding matrix is PϵQ mxn. Let “P” and “Q” stand for LSTM and embedding dimensions as the logistical sigmoid initiation.

**Figure 1:**
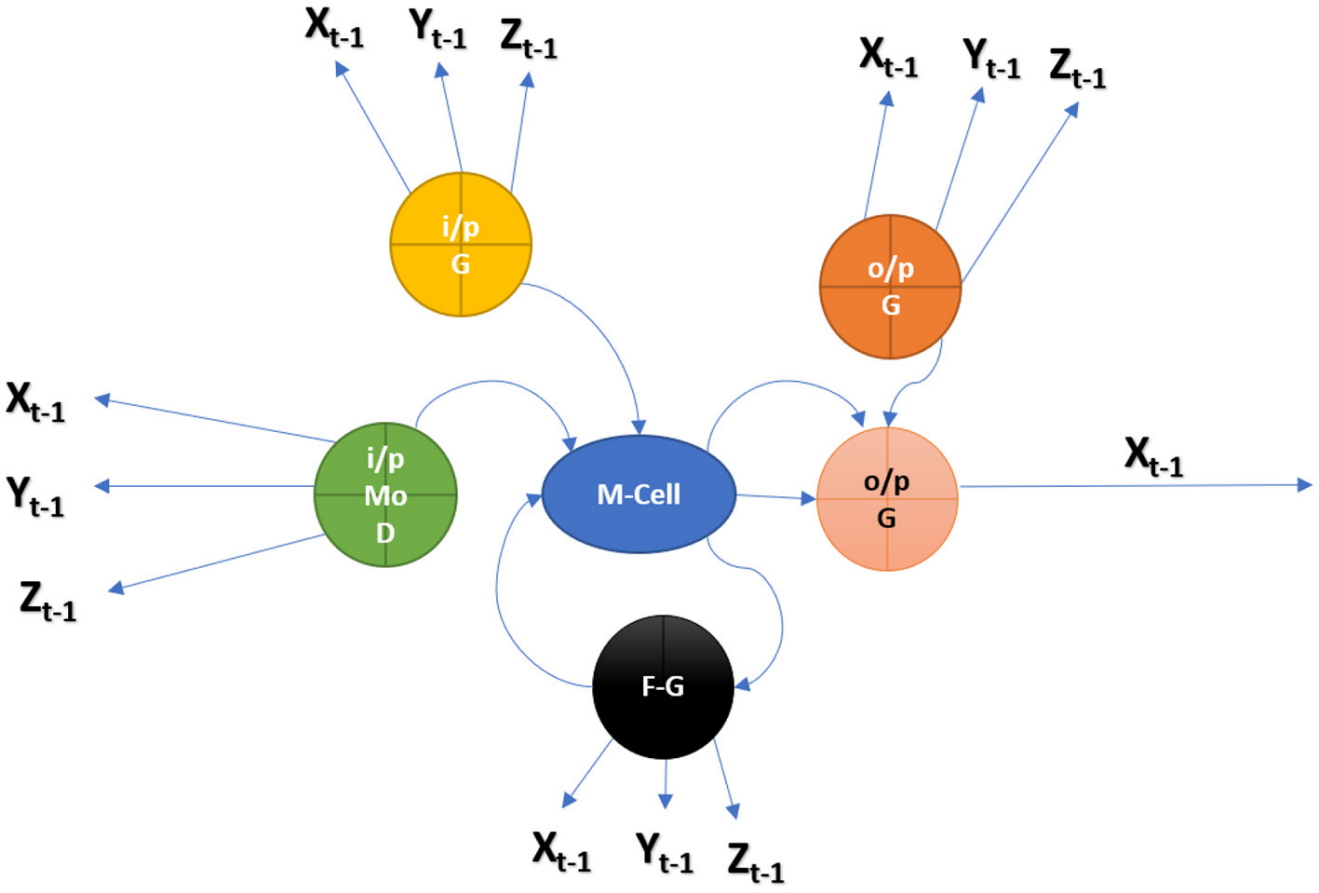
Cell format of LSTEM.

Figure 1 shows an LSTM cell, along with bolded squares indicating projections of the learned weight vector. Every cell learns how to balance its input components while also studying how to control its memory supply. Also, it learns weights that delete the memory cell as well as weights that govern the emission of memory. The Context Vector (CV) is a dynamic depiction of a related component of such visual input to a given point in time (*t*). By defining a method, “ϕ,” which calculates CV beginning Annotation Vectors (AV) ai, I = 1,…, loc corresponding to such features obtained at various picture settings. The mechanism generates a positive weight I for each location “loc” that can be taken as whichever is the probability of such location “loc,” it remains the best place to develop and to generate the succeeding word (stochastic attention method) or else the virtual importance to bounce to location “loc” in merging the αi's (αi's blending mechanism) (23). Non-linear and linear data can be learned by NN. Where the neurons are thought to consist of multiple layers. An MLP is a directed graph of input nodes that connects the input and output layers. As a supervised learning technique, backpropagation is used to train the NN in this case. An attention model, for which we apply a multilayer perceptron conditioned here on preceding HSt−1, computes the weight αi of all vector annotation AI. The HS changes to be the output of RNN that progresses through their output sequence: “here” the next network search is determined by its previous series of words (Figure 1).


(4) $$\begin{array}{r} \begin{array}{c} HS_{t}=f_{ATTN}\left(ai,HS_{t-1}\right)\\ \alpha_{t}=\frac{Exp\left(HS_{t}\right)}{\sum_{n-1}^{Loc}Exp\left(HS_{i}\right)} \end{array} \end{array}$$

From Eq. (4), the CV (St) is calculated after the weights have been computed, while “ϕ” is stated as a function, which gives a specific vector based on a sequence of AV as well as its related weights. An average of such AV fed *via* two distinct MLPs (Intc, Inth) predict the LSTM's initial memory state as well as HS, Eqs (5), and (6)


(5) $$\begin{array}{r} S_{t}=\alpha \left[a_{i}\right],\left[\partial_{i}\right] \end{array}$$

(6) $$\begin{array}{r} S_{0}=F_{\mathrm{int}},a\left(\frac{1}{loc}\overset{loc}{\underset{n}{\sum }}a_{i}\right),h_{0}=F_{\mathrm{int}},h\left(\frac{1}{loc}\overset{lco}{\underset{n}{\sum }}a_{i}\right) \end{array}$$

An average of AV fed into two independent MLPs predicts the LSTM's baseline memory state and not the HS (Intc, Inth). For calculating the output word probability in this study, we are using a deep output layer. Cueing out from the image, the predefined word, as well as the Decoder State are all used as input (DSt), Eq. (7)


(7) $$P(Y_{t}|a,Y_{1}^{t-1})\equiv Exp(loc_{0}(E_{t-1}+L_{t}DS_{t}+L_{t}R_{t})$$

### Named entity recognition using LONIC

**Figure 2:**
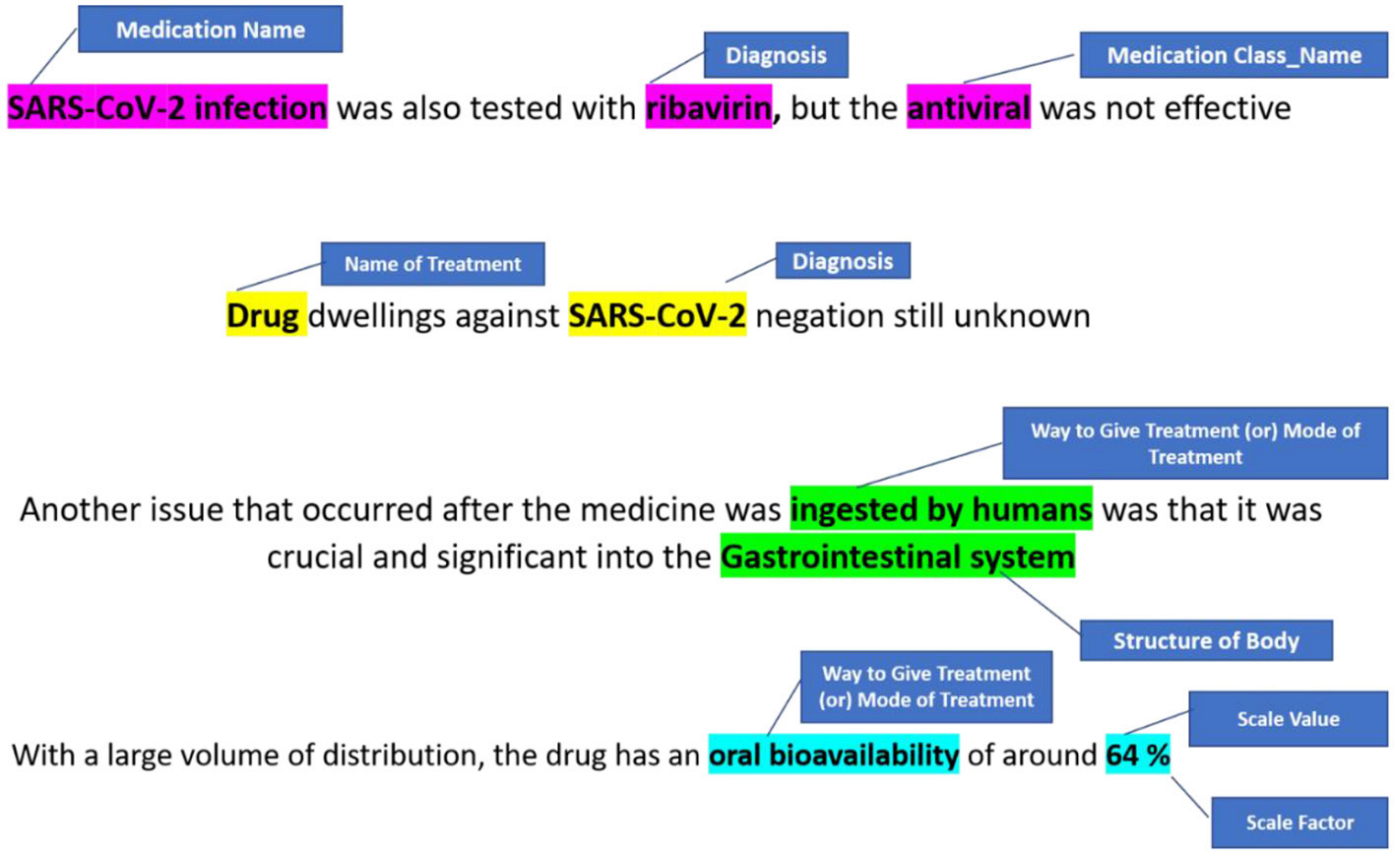
Example of NER for clinical symptom extraction.

We designed a rule-based approach to automatically normalise a local testing name towards a Logical Observation Identifiers Names and Codes (LONIC) code of MedIMG utilising data from several medical systems. Following tokenization of the input lab testing names, specific items are recognised, and relevant LONIC codes get automatically mapped depending upon that coding rules (24). Figure 2 depicts an overall view of the MedIMG TestNorm system's module, which primarily includes NER as well as LONIC map-based modules, using inputs from lexicons/coding rules.

NER is a text processing technique that recognises words belonging to specific Named Entity categories (25). This is a crucial tool in NLP to extract information from documents. The network is trained to self-learn subject-related terminology with the aid of a dense architecture with DL models. All named entities inside the document are identified by NER. The NER annotator, in particular, uses multiple ML algorithms that can tag entities using standards for identifying numerical entities such as time/date formats. The goal of NER is to find the symptoms that are associated with the sentences collected from EHR system (26).

Logical Observation Identifiers Names and Codes is a database with a vocabulary coding system designed to make detecting and presenting medical laboratory observations more uniform and global. Component, System, Method, Time, Property, and Scale are six fundamental axes used by LONIC to characterise each notion (27). Nine of these were included with our MED IMG entity classifications. Component, System, Method, and Quantitative/Qualitative define whether a test produces a quantitative/qualitative result, and Institution, that specifies a testing kit maker is our five root groups (28). The entity recognition process is divided into two phases: (a) a preliminary phase, which integrates dictionary lookup with regular pattern matching, then (b) a class of activities phase that translates unclear tags from preliminary step to final tags using a set of predetermined rules (Table 1).

**Table 1:** Summary of database and LOIN code.

**Database type**	**Meaning**	**Abbreviation**	**Full name**
Characteristic	Measurement component/anion	Acnc	The surface of the hepatitis B virus
Area	The type of measurement; includes a conceptual model (dosage, composition, factor, subset, and exceptional)	P_t_	Single point time
Monitoring	Identifies the testified measurement's time things, which are treated as a SPT	Ser/Pls	Plasma vs. serum
Method	It refers to a set of data/specimens being measured	Q_n_	Qualitative research
Level	Qualitatively channeling's the monitoring value	IA	Biosensors based on metabolites
Procedure	It involves the procedure used to extract the standard size		

**Figure 3:**
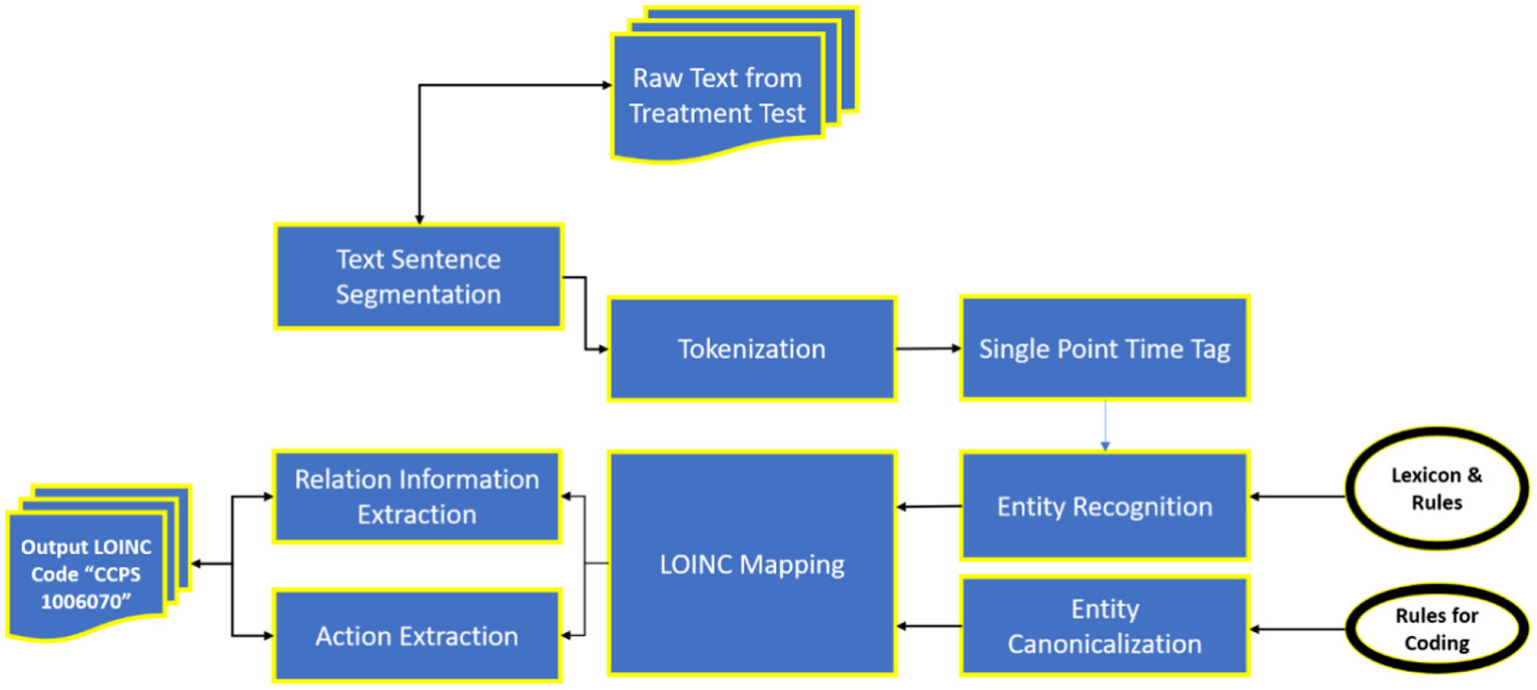
Mapping of LOIN codes and rules.

Many data can be recorded and categorised with their appropriate category within the first stage, but some problematic words will have to be examined further. For instance, the letter “IA” could be mapped to either a “method” and is the abbreviation of “immunoassay” or a “system,” which is the state of “Iowa.” To select the relevant semantic classifications for such phrases, we built context-based criteria. Such investigation code must be now a LONIC code so that an identifier can be sent. A LONIC code (Figure 3) may be provided additionally towards a legible registered displaying name, which correlates for specific internal purposes to the degree permitted by the protocol. These characters can be classified with high accuracy because they have a range of data about the flow of the text being written. This makes it simpler to distinguish between different characters in the text. After the raw text has been preprocessed, a feature extraction step is performed to determine important details about each character, such as loops, quality of decision making, aspect ratios, etc. A proposed model is then used to analyse these features. Due to the manual step of extracting features and the fact that DL models can only do so much, their performance is not very good. In this section, we analysed the data to determine whether the researchers should revise certain sections of the paper. Tables listing handwritten names, layout, results obtained, and remarks are preferable because most empirical studies use the same algorithm with comparable performance datasets.

The use of LONIC across many application fields is becoming progressively acknowledged worldwide over the last 25 years (29). According to the Regenstrief Institute's LONIC webpage, there were about 85,000 clients of LONIC in over 176 countries, and LONIC is becoming the “lingua franca for medical data interchange.”

### Construction and structure of LONIC

**Figure 4:**
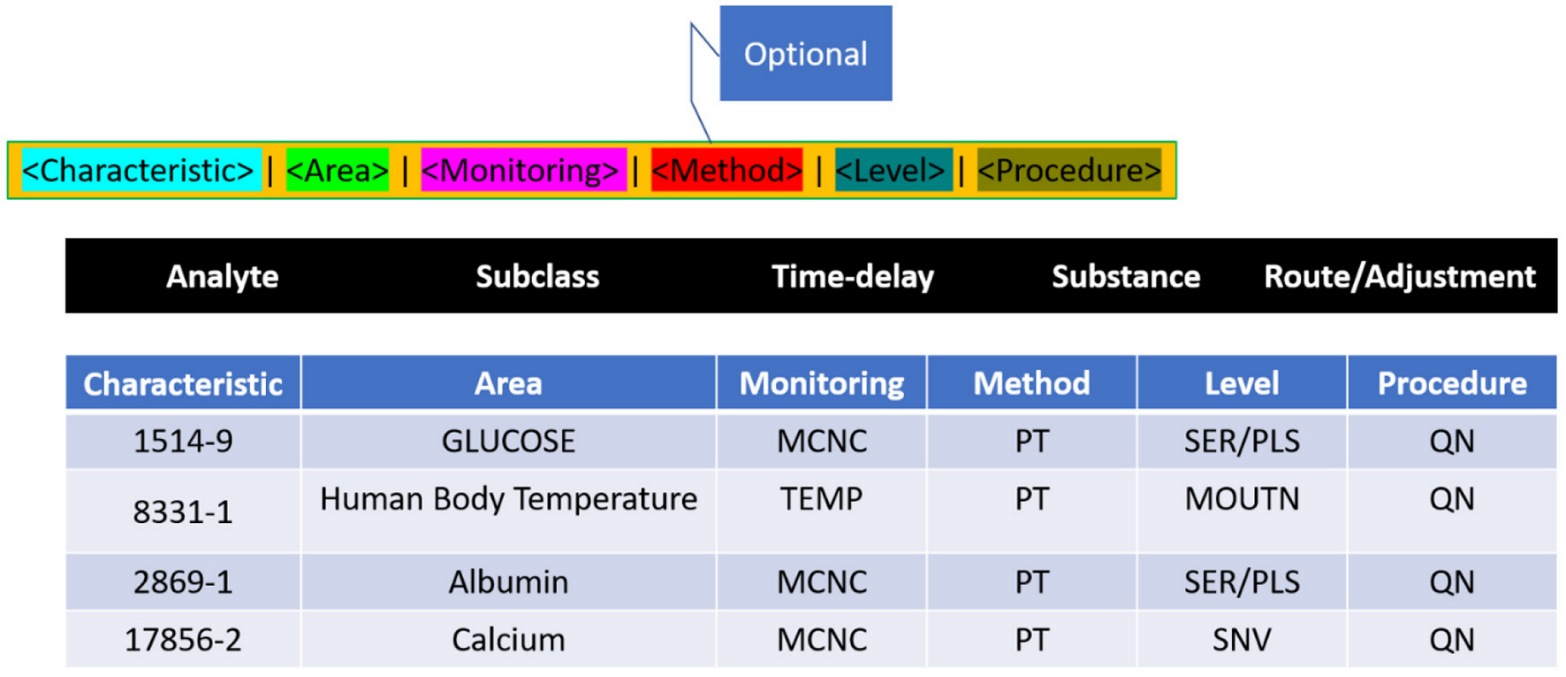
LONIC nomenclature.

Whatever the laboratory test, medical observation/clinical examination is clearly determined in Terms of six axes, as per the following LONIC concept (Figure 4): Component, Property, Time Aspect, System, Scale, and Method Type. The above six axes are given in the LONIC database by corresponding columns (30).

**Figure 5:**
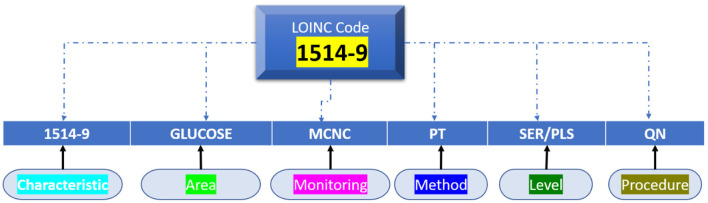
Quantitative measure of LONIC name and code.

The technique of investigation in the sixth axis is limited in that it must be specified as required for precise determination to assess the examination under issue, and this differentiation is crucial in clinical practice. As a result, this column is frequently empty. All six axes add up to the unique, complete generic identity for the examination, as per the LONIC nomenclature guideline (31). Each form of investigation, measurement, or observance, which varies in any of these six axes, is assigned a unique LONIC code, as well as an individual entry inside the LONIC database. These LONIC codes are being posted sequentially to the access; unlike a categorisation, the codes need not indicate links within concepts. It is made up of a numeric multi-digit code of only a check digit at the end that is generated using the Mod10 method (32). The process is documented inside the LONIC User Manual primarily by Regenstrief Institute and the LONIC Board (33) (Figure 5).

Towards summation, all medical adherence/clinical assessment is represented with seven axes: the previous LONIC encryption as well as the recognised name, which consists of the six axes defined on top. Figure 5 shows the example of measurable urine sugar quantity using test strips.

The probable discrimination between purposes of the similar chemical, such as glucose, with dissimilar test materials like urine, cerebrospinal fluid, or serum, as well as through different procedures, scaled type, also attributes, occurs using the six axes (34). Only the property is clearly defined on the Property axis, not the allocated measuring unit and its representation. To accurately attain the specific LONIC code for such an evaluation issue, it is vital to recognise and describe all six axes. By taking a glucose amount, for example, that is shown by determining [Property = SCNC], a molar substance absorption would be distinct from determining a mass absorption [Property = MCNC]; collected results will obtain different LONIC codes in a similar example category. However, whether the LONIC code is represented in component g/L or mg/dl, and specific components are being stated at machine-readable procedure, it has no bearing on the code's uniqueness (35).

## Proposed methodology

### Investigation of clinical texts for information retrieval

An end-to-end “pipeline” for obtaining crucial clinical information from narrative texts is being designed. Negation detection is used to filter these features, and the residual features are mapped to SNOMED-CT terminology.

**Figure 6:**
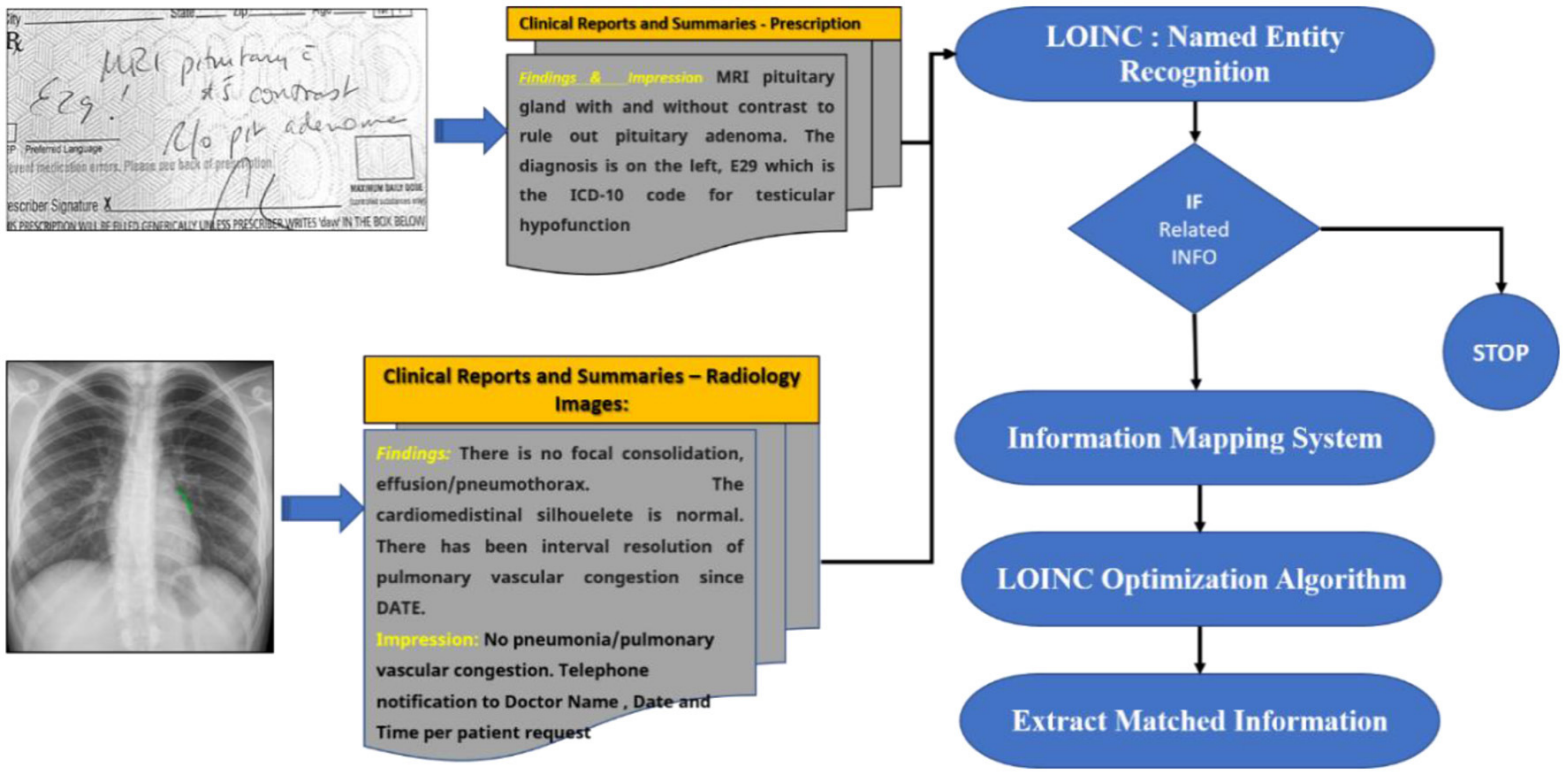
A proposed model for extracting patient treatment features from MedIMG.

A feature extraction pipeline of clinical text is depicted in Figure 6. The TextRank algorithm (36) is used to implement the content summarising module. Primary clinical information such as problems, procedures, and tests are extracted using the CLiNER concept recognition algorithm. The features inside a negated context are subsequently filtered using an updated Negex algorithm. This part of the pipeline is a secure in-house online application that allows users to submit files that contain descriptive clinical notes and extract essential clinical features together with their contexts. From this, you can pursue one of two paths: (1) Use MetaMap to map the retrieved features towards the SNOMED-CT terminology systems and filter out unmapped features. Eliminate standard terms (e.g., “Human Body Structure,” “Clinical investigations,” “Biological agent”) from the top two levels of the SNOMED-CT concept tree using the hierarchical system of SNOMED-CT and MetaMapLite; (2) use the terminology mapping tool formulated to map those particular concepts to SNOMED-CT effectively. These standardised concepts can be aggregated in input features that can be directly fed into knowledge discovery ML algorithms. We illustrate the usage of EHR information extraction strategies for several patients in this section (37).

### Automatic classification of MedIMG by image resolution

The IAM Handwriting Database is applied to arrange the handwriting, as previously stated. To begin, everyone must first register on the website and retrieve a folder of photos as well as XML files containing the text location in the image. In this study, N's do not require significant pre-processing of raw data (33). As a result, it will not make any changes or adjustments in these images but rather send the entire image and just patches of text to the NN.

#### Generating patches of data

**Figure 7:**
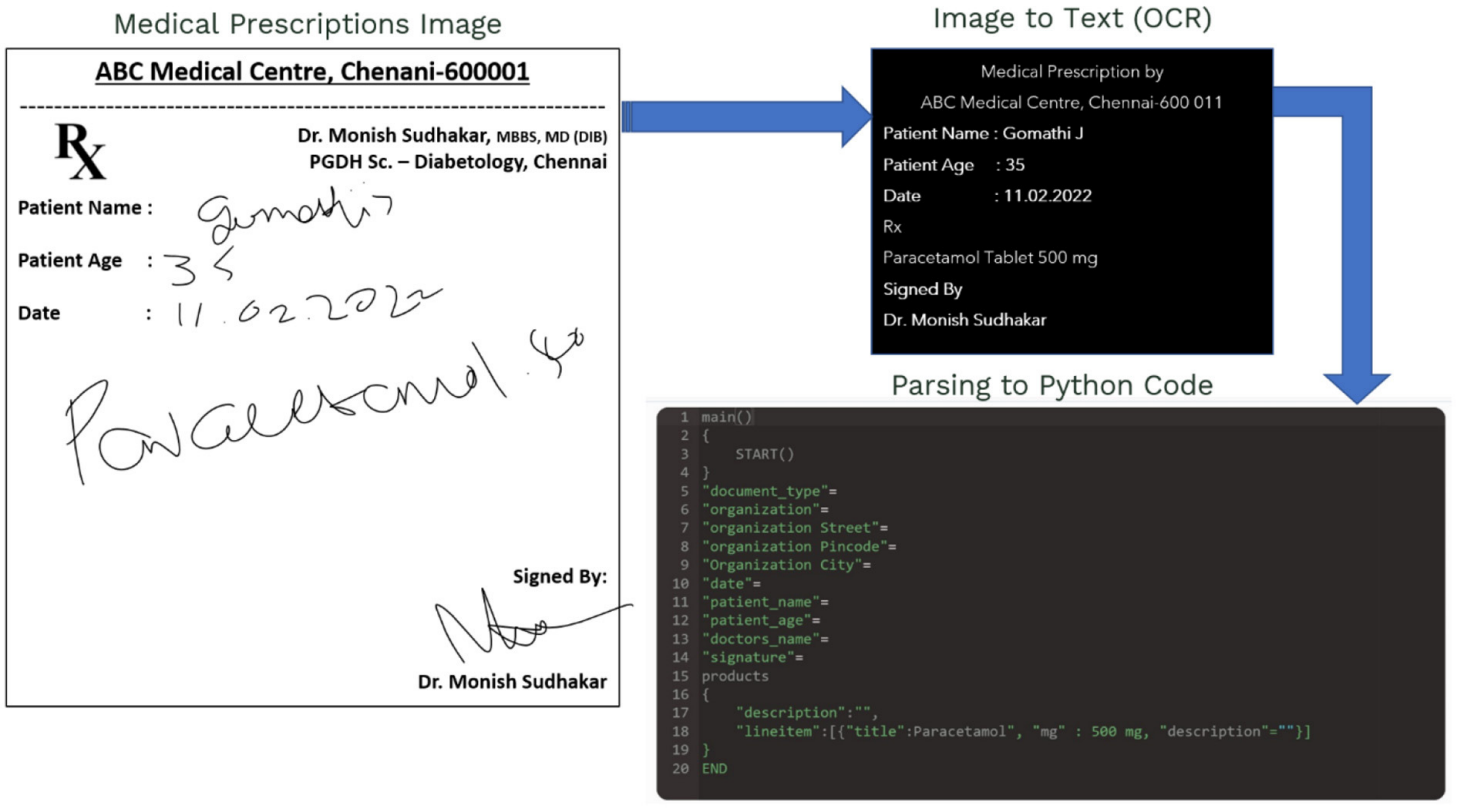
Cropping and generating random patches of MedIMG.

Instead of transferring specific sentences or words, merely random patches containing text are allowed to train the NN that comprehends the writing style of doctors. Cropped photos with a random size of 113 × 113 can be used to generate patches from each sentence. Figure 7 below is a mosaic of eight of these patches.

A simple generating function is constructed using Python to traverse over each text, allowing for cropping as well as generating random patches for images. We can limit the number of patches caused for each image to 70% of the total generated patches.

#### HWNet v2

HWNet v2 is a new version of the HWNet network. We restricted the number of convolutional layers in our basic HWNet architecture to five, which is equal to such suggested layers at AlexNet. Innovative structures, like VGGNet, GoogLeNet, as well as ResNets, have recently demonstrated deeper CNN networks enabling greater performance but more discriminative features. The following are some of the important architectural innovations used in such networks that result in more efficient training: (a) using lower-dimensional filters (3 × 3), thus taking fewer parameters with large size filters that also perform as an essential regularizer, (b) by using (1 × 1) filters that perform as dimensionality lessening unit to retain the no. of parameters in rheostat, (c) by using inception layer that presents multi-scale functioning to have several equivalent layers functioning with different scales, and (d) using remaining blocks for studying remaining function *F*(*x*): = *H*(*x*) – *x*. Here *H*(*x*) is anticipated for underlying mapping. The remaining layer is usually applied with a crosscut linking in the absence of available parameters.ResNet34 network of four blocks, each containing several ResNet modules in our upgraded HWNet architecture, dubbed HWNet v2, is used. Rather than employing globally averaged pooling, we discovered completely connected layers towards the conclusion of the process to learn superior features from the penultimate layer. The HWNet v2 network configuration is summarised in Table 2. Every ResNet module has two convolutional layers in it.

**Table 2:** Layer configuration of HWNet v2.

**CNN**	**L-1**	**L-2**	**L-3**	**L-4**	**ROI**	**FCNN**
3 ×3	[3 ×3,64] ×3	[3 ×3,128] ×4	[3 ×3,256] ×6	[3 ×3], 3	[6 ×12]	[2,048] ×2

#### DenseNet models

The 121-layer DenseNet model is chosen as the basic model in this investigation since it produced the best results in most of the previous experiments on CXR classification. This model was initially trained by scratch, but we were able to improve its performance by using pre-learned weights using ImageNet. Binary Cross-Entropy (BCE), a multi-label loss, is used to train the model. We use the ImageNet dataset's mean as well as a standard deviation to normalise all the images. We apply data enhancement techniques like random horizontal flipping as well as uniformly random rotation from −15° to 15°.

To various image sizes, the Adam optimizer is applied at multiple primary acquiring rates. To varied image dimensions, the learning rate is measured by the actual batch size, which is shown in Table 3. If a validation loss does not reduce across three epochs, the learning rate decay is doubled to 0.5. If such a validation deficit cannot increase after ten epochs, the test must be terminated. Rather than using the MIMIC-CXR-JPG dataset's official train validation-test split, five-fold cross-validation is applied to 20% of test data from a dataset. The residual 80% of the data is being split as 90–10 for training validation purposes.

**Table 3:** Classification of learning rate.

**Size of**	**Rate of**	**Group size of**
**input image**	**learning**	**input image**
256 ×256	0.0051	512
512 ×512	0.00034	256
1,024 ×1,024	0.000181	180
2,048 ×2,048	0.0000918	75

#### Effective receptive field

An effective receptive field (ERF) comprises the centre pixels inside the receptive field that have the maximum effect on the results. The impact of distribution inside the receptive field becomes asymptotically Uniform, according to Luo et al., and also, the effectual receptive field is just a segment of a theoretic receptive field. Moreover, as the network is taught, an effective receptive field grows larger. For calculating the ERF, a trained model is uploaded, run in evaluating manner, enters the data, and then averages the square gradients to get the uncentered sample variance. We use the centre pixel and backpropagation to generate one gradient per image. When computing the ERF, we also add huge random translations to the data. The value for image Xi at pixel (P, Q) is referred to as Xi (P, Q), as well as channel 'n', and pixel (*x, y*) of a NN's final convolutional layer activation is referred to as Fn (*X*_*i*_, *x, y*). An ERF is a scalar image whose value shows the contribution of every pixel *X*_*i*_ (*P, Q*) to such activation. The partial derivative is used to calculate the impact, Eq. (8)


(8) $$\begin{array}{r} \frac{\alpha F^{a}\left(X_{i},\;x,\;y\right)}{\alpha X_{i}\left(P,\;Q\right)} \end{array}$$

This derivative is determined not just by the NN's weights but also by the input image *X*_i_. Back-propagation is used for quickly calculating the partial derivative, Eq. (9)


(9) $$\begin{array}{r} ERF\left(P,\;Q\right)\;=\frac{1}{p,\;q}\overset{p}{\underset{p-1}{\sum }}\overset{q}{\underset{q-1}{\sum }}\frac{\alpha F^{a}\left(X_{i},\;x,\;y\right)}{\alpha X_{i}\left(P,\;Q\right)} \end{array}$$

where “*n*” denotes the number of links inside the network's final convolutional layer, and (*x, y*) denotes the central point for every image *X*_*i*_. Since backpropagation gradients are positive and negative, first square them and then find the average for avoiding cancellation, but the authors of the original ERF study do not square the gradients while averaging.

**Figure 8:**
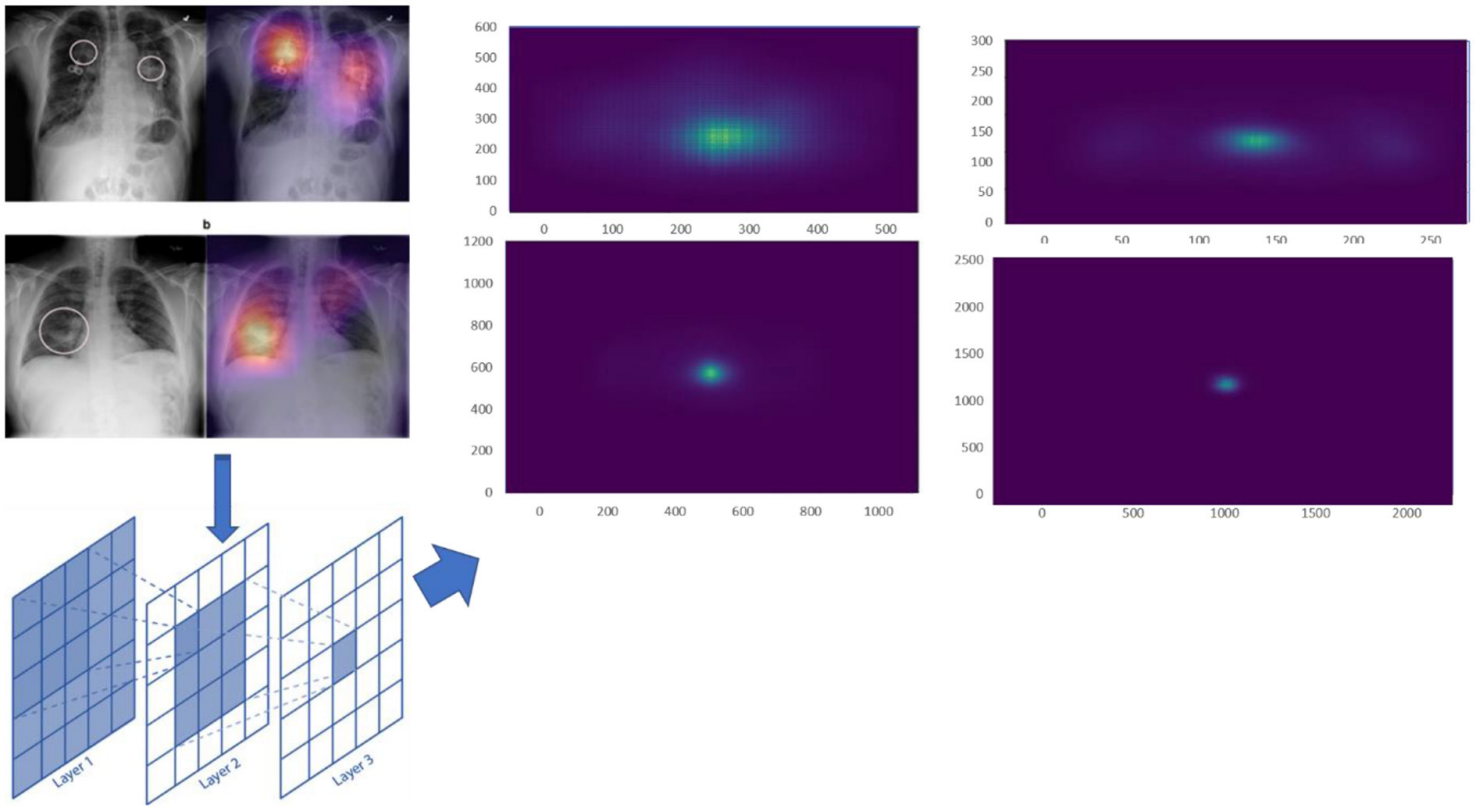
A trained Dense'et121's ERF.

For various scale input images, we calculated the ERF from trained models. The ERF of DenseNet121 introduced models with different resolution photos is shown in Figure 8. With increased picture resolution, the ERF reduces in size in relation to the source image. When using CNNs upon high-resolution images, it suggests that large receptive fields are required.

#### Stacked scale-CNN model

**Figure 9:**
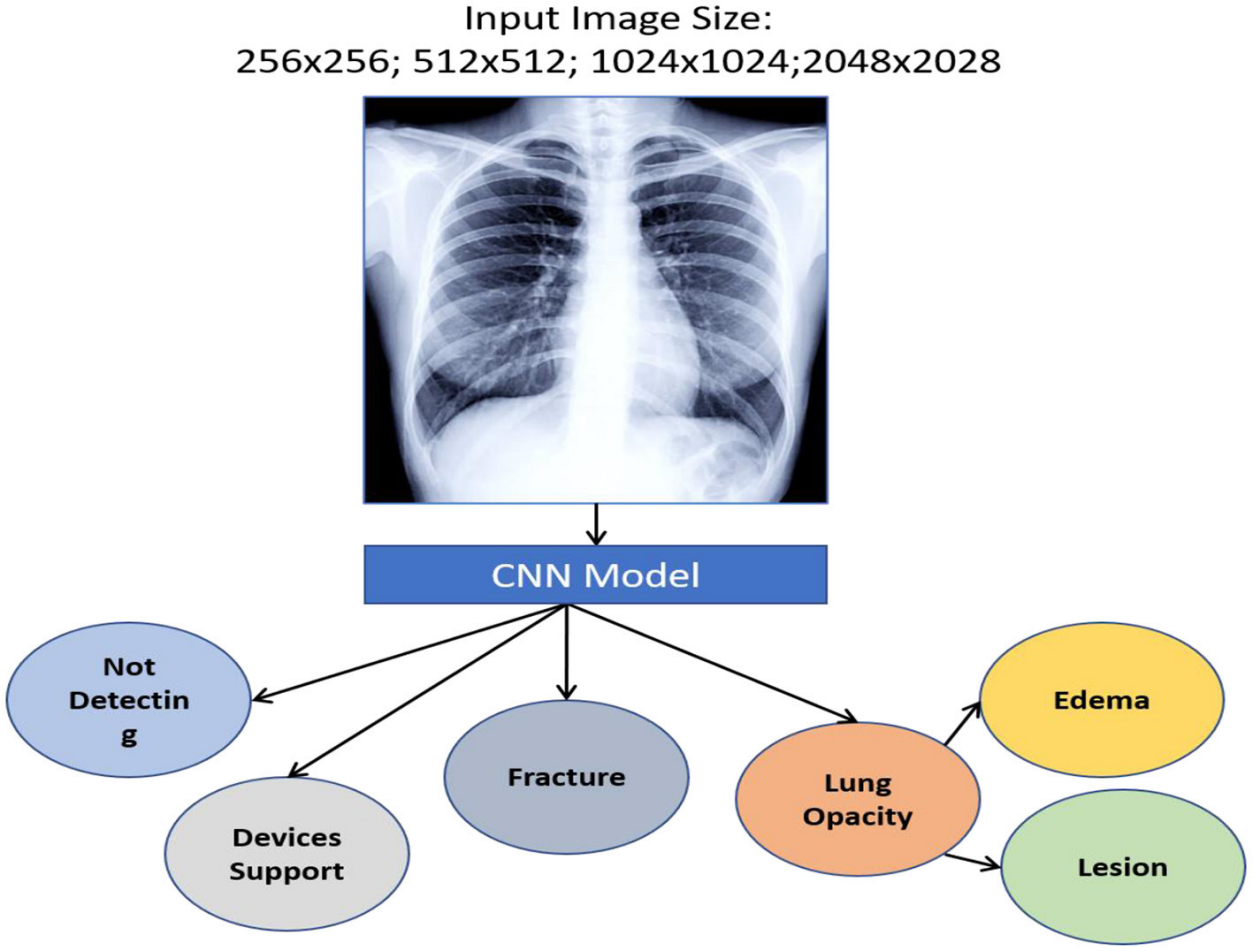
Workflow of CNN-based MedIMG.

We used a stacked CNN model in which discrete image scale sizes (256 × 256, 512 × 512, 1,024 × 1,024, and 2,048 × 2,048) are used to train the four models to enhance efficiency while keeping the flexibility for using pre-trained models. We train a specialised CNN model for deriving weights to each scale using the concatenation label probabilities of validation data from all these four classifiers providing features for a specific label. The CNN approach is depicted in Figure 9.

The weight to finding (F) and scaling (S) is wfs in our model of multi-label stacked CNN that possesses a single parameter P∈R14 ×4, which is a 2-D tensor. In order to prove that a given label amount is one for the acquired weights of such four scales, we apply a SoftMax parametrization, Eq. (10)


(10) $$w_{fs}=\frac{Exp(V_{fs})}{\sum_{i}^{}Exp(V_{fs})}$$

For each finding f, its parameterization satisfies ∑s *w*_fs_ = 1. By defining *v*_fs_ = 0, we make the weights *w*_fs_ constant. These weights will then be used for averaging the probability of each scale.

**Predicted label (PL):**


(11) $$PL_{f}\left(x_{i}\right)=\sum_{i}^{}w_{fs}P_{fs}\left(x_{i}\right)$$

We perform a numerically consistent calculation of ∑s *w*_fs_σ(PL_fs_(*x*_*i*_)) for each finding (*F*) for an image input of *x*_*i*_ with PL_fs_(*x*_i_), in which σ(*z*) = (1 + Exp(–R))1 is the sigmoid function. Although all labels are calculated simultaneously in our approach, each label “*F*” is processed separately under this proposed model.

This stacking EM is trained using the validation data's output log produced by four classifiers with the help of four scales. The model is trained across 100 epochs with a 0.1 learning rate that decays exponentially, guaranteeing the converging of weights. For acquiring the ensemble probabilities for each label, multiply the learned weights by this model using predictions generated from test data.

#### Lion optimization algorithm

The lion optimization algorithm (LOA) is a bio-inspired algorithm that imitates lions' basic personality. Lions are naturally socialised, which causes them to divide into rover and habitant lions. A grown male lion is usually removed from the pride, and now these lions have no role in the pride, thus making them rovers. The rover lion can attack the lions inside the pride at some point in order to join the group. As a result, the LOA looks for rover lions and evaluates their quality. Fitness evaluation is the name given to this type of quality assessment, and only the fittest lions/lionesses are allowed to join the pride. The lions with lower fitness levels are omitted from the evaluation process. This cycle is repeated till better solutions are found. A certain percentage of lions are designated as rovers, while the others are assigned to points of pride. The gender ratio, as well as the border limit for each pride of lions, have been established.

The main key points of LOA are:

- In order to join the pride, the rover lion attempts to defeat the resident lion at any given time.
- The pride's fittest lion is the only one that can survive, and the rest unfit lions are ignored.

The overall number of points of pride remains constant, and rover lions have a chance of finding a proper fulfilment with a probability of [0,1].

Taking such benefits into consideration, this work uses LOA to determine the ideal filter order to denoising the image. When a filter is applied to a picture, the image's fitness is evaluated. The Peak Signal of Noise Ratio (PSNR) value is used to calculate the fitness value of such a work. The second filter is applied to the image once more, and the fitness value is checked. This method is repeated until the image does not quite improve for three consecutive rounds, at which point it is regarded as the best response. The following is the algorithm for the suggested task.

(A) Proposed Lion Optimization Algorithm

Step 1. The input of Noise-filled picture

Step 2. Denoised output Images

Step 3. Begin

Step 4. Establish a starting population of lions and prides

Step 5. Do

Step 6. Assess rover lions' matching;

Step 7. If matching (New)> matching (Old)

Step 8. Replace matching (Old);

Step 9. While (close condition);

Step 10. End

The PSNR value of an image is used to calculate image fitness, which analyses the quality between source and denoised images. The following Eq. (11) is used to calculate this.


$$\begin{array}{r} Suitable=10 \end{array}$$

(12) $$\begin{array}{c} \times \log_{10}\frac{512\;\times \;512}{\frac{1}{hxw}\sum_{a-0}^{h-1}\sum_{b-0}^{w-1}\left[F(a,b)-G(a,b)\right]}DB \end{array}$$

The height, as well as width of such images, are represented by *h* and *w*. The grey levels of the original MedIMG and denoised image at the (*a, b*) position are *F*(*a, b*) and *G*(*a, b*), respectively. The trial-and-error method is used to select all of the work's beginning settings. The PSNR value is used to calculate the fitness, which allows the image quality to be evaluated and processed appropriately.

### Proposed hybrid model to predictions of RNN (HWNet v2) + LSTM + GRM

The next character from MedIMG is predicted using an RNN. RNN's LSTM cell is used in this study. Each time LSTM processes a word, the following letter in the drug's name is determined by computing the probability of such possible values. The memory state of the network is initialised with a vector of '0's. So that it may be readily updated when each word has been scanned. The “x” in Figure 2 represents a feature, while the “o” represents a vector of size length probabilities. Because of their sequential structure, RNNs have gotten much attention for EHR predictive modelling. RNNs are Artificial Neural Networks that have recurrent connexions in the hidden layer. The HS of “ht” is updated successively based on activating the current input “xt” at a time “t,” as well as the last HS of the layer ht−1. The Gated Recurrent Unit (GRU) and LSTM models are two popular RNN versions. The necessity for these variants arises because of the problems encountered while training traditional RNNs, where gradients would frequently vanish. Two major approaches have been investigated to address the vanishing gradient problem for extended sequences: (a) Develop an alternate training algorithm to the stochastic gradient, such as a clipped gradient. (b) Contain an internal cell state which controls the information flow *via* a series of gates in conventional RNN units. LSTMs and GRUs, unlike typical RNNs, have a recurrent internal loop at every unit and also with three and two gates to regulate information flow. Data have demonstrated that gated RNNs can identify long-term dependencies as well as avoid the problem of vanishing gradients.

**Figure 10:**
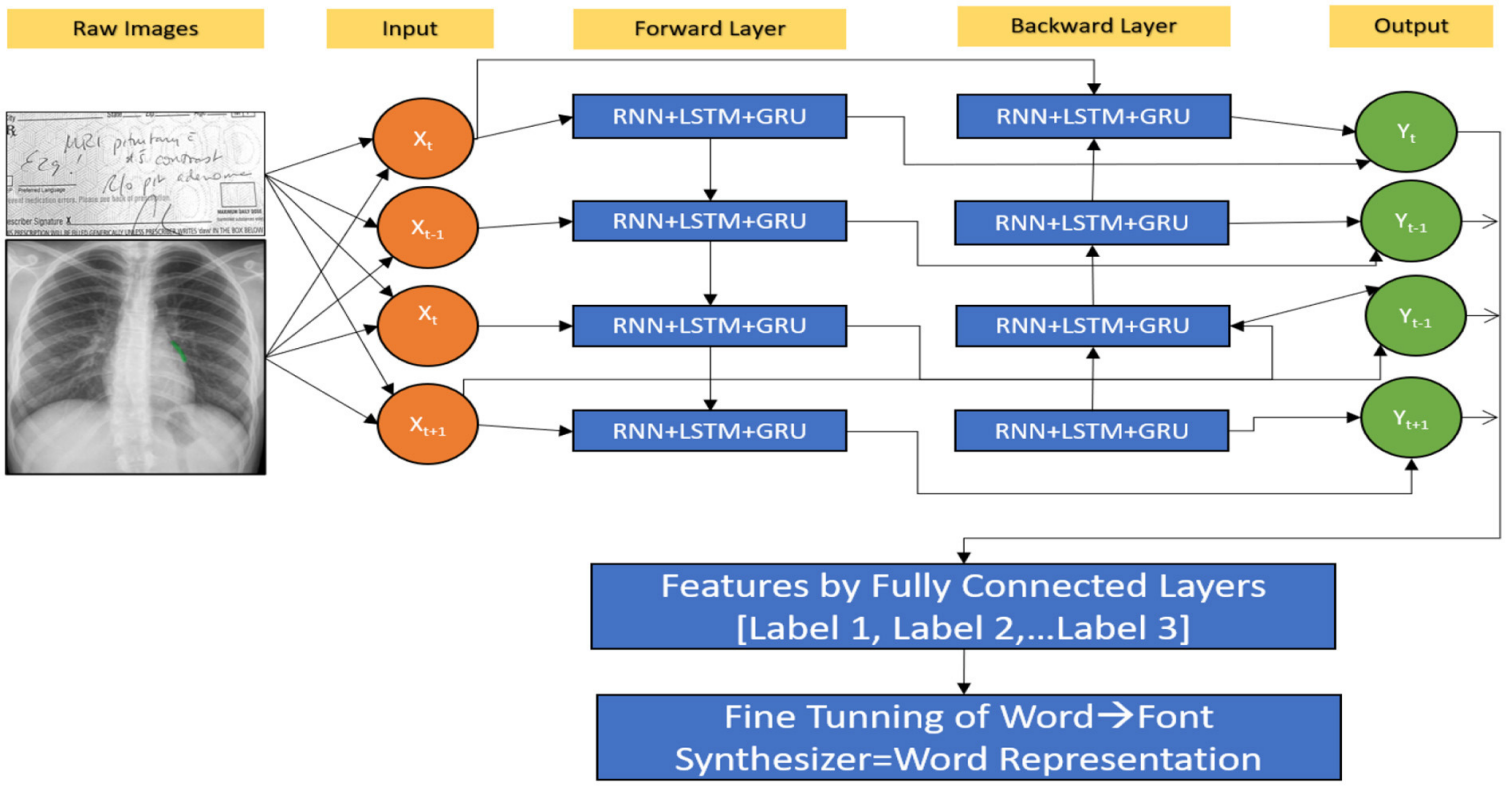
Hybrid RNN (HWNet v2) + LSTM + GRU.

A patient in an EHR is made up of a series of visits, and if each visit is represented as a numerical vector, those vectors can be fed into the RNN model in order to predict the desired outcome. In this study, we predominantly used the LSTM network to be the prediction model (Figure 10). The model recognises one visit for each time step of a patient “p” with “Tp” visits. From *t* = 2 to *t* = Tp, the new LSTM state is determined by the previous time step's state as well as the new input visit. The LSTM block spreads its state to subsequent dense layers at each time step. This indicates that the network has an input and output at each stage. Sequence-to-sequence prediction is the name given to this LSTM arrangement. We explored the multiple baseline prediction models, including several ML methods and LSTMs.

#### NER-based open-source UMLS

**Figure 11:**
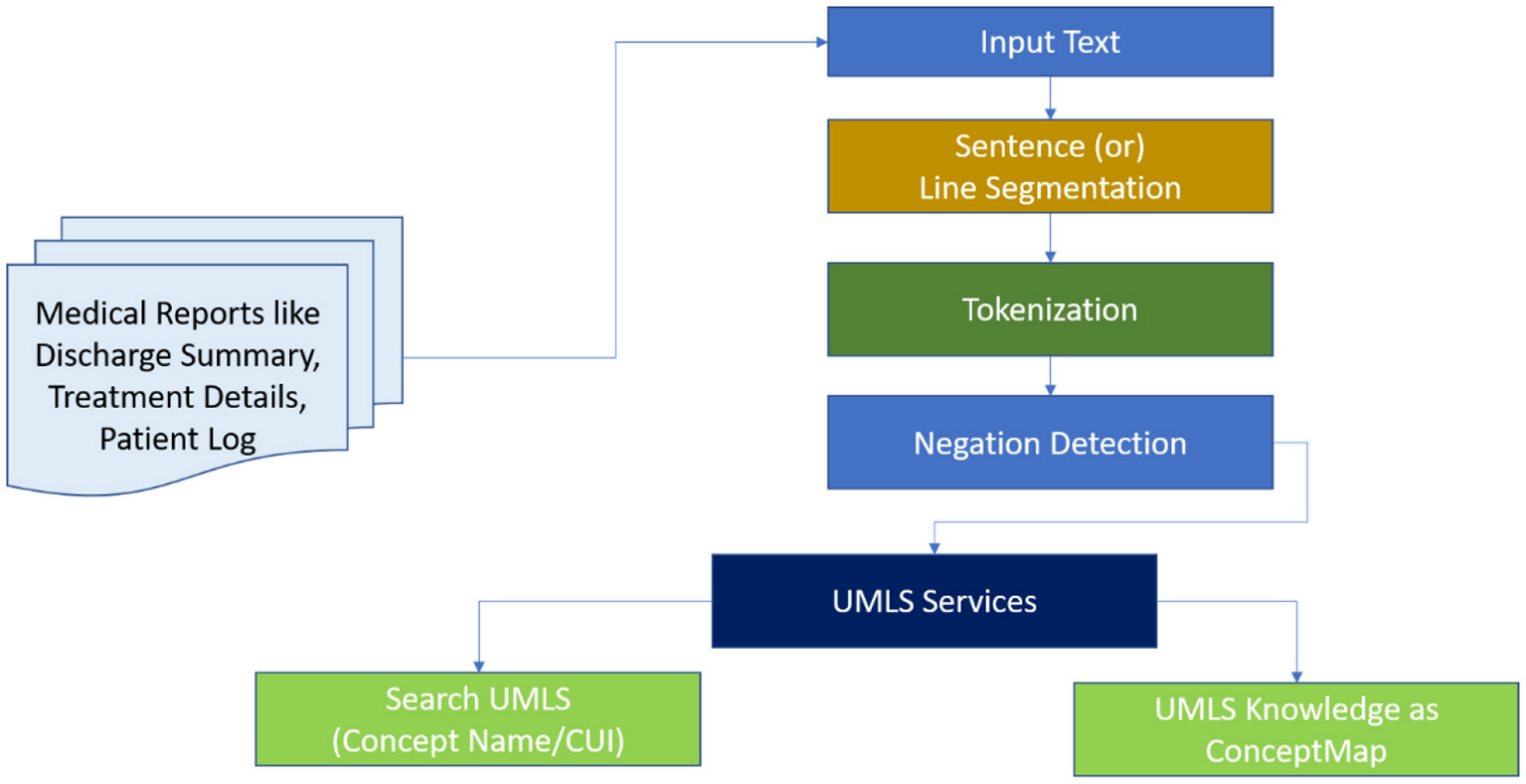
MetaMap lite procedure.

MetaMapLite leverages ConText19/NegEx13 to negation detection. Consequently, the users who supply the longest concept appropriate to the semantic type of UMLS are provided. The processing pipeline is divided into seven steps, as shown in Figure 11 and detailed below.

Either the default OpenNLP sentence segmenter12 or its blank line segmenter is used for sentence/line segmentation. The basic MetaMapLite tokenization algorithm is used for tokenization. The part-of-speech tags that are optional are done by default OpenNLP part-of-speech tagger. The predefinition of the token window occurs for paralleling the sentence length using the OpenNLP segmenter; on using the blank line segmenter, a nonoverlapping window with 15 tokens occurs. There is no additional chunking. With minor modifications, term normalisation is based on MetaMapLite string normalisation. Before a dictionary lookup, the following procedures are performed on a term: (a) Parentheticals are removed, (b) syntactic inversion is performed, (c) lowercase is converted, and (d) possessives are stripped.

**Figure 12:**
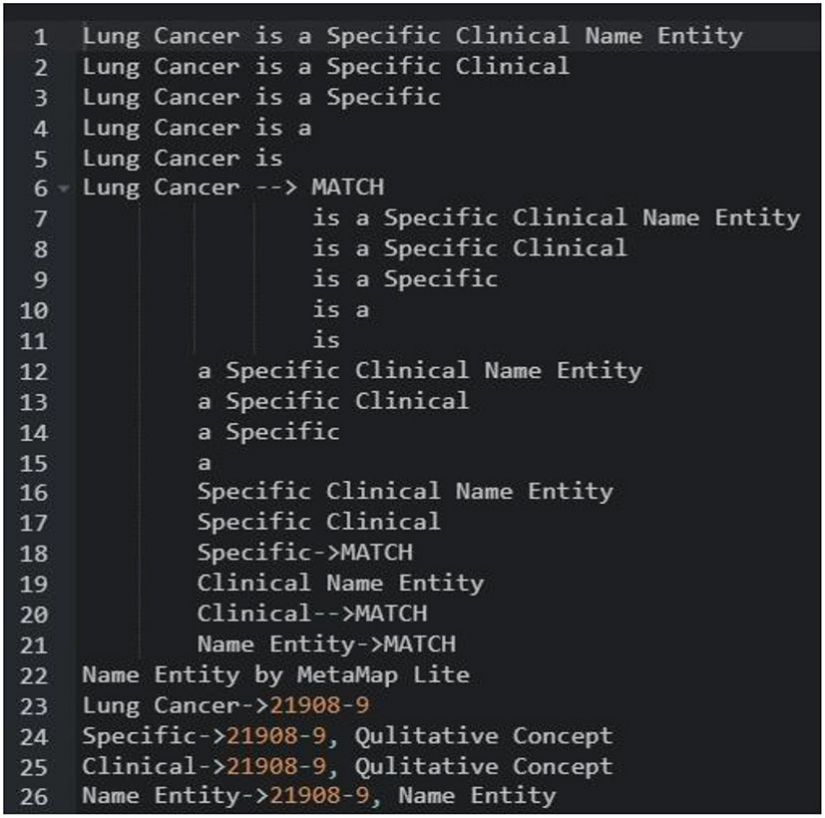
Test finding of UMLS-based NER.

As shown in Figure 12, mapping is done to sentence/line-based chunks that are dynamically separated into sub-lists during processing. Each piece is normalised before being compared to a dictionary. Any dictionary match that is obliterated by a lengthier match gets discarded. MetaMapLite presently uses three dictionaries generated specifically for MetaMapLite for dictionary lookup: (1) CUI Concept, which maps CUIs to concept predefined names; (2) CUI Source Info, which holds the UMLS CUI, the UMLS string ID, a sequence ID, a source-derived string ID, a source abbreviation ID, and also a source term type ID; and (3) CUI St, that maps CUIs into semantic types. As an example of sentence-level NER, a token list for the phrase “Papillary thyroid cancer is a unique clinical object” is initiated and filtered.

## Experimental results

The accuracy of a model can be improved by training it using a vast amount of data. As previously stated, we train the model using all generated data. TensorFlow's training is divided into two categories: iteration and model during training. We apply the TensorFlow standard model limiting iteration to around 10,000 for this research work. The dataset of MIMIC-CXR-JPG that was used in this investigation is discussed. It is a big one, comprising 377,110 X-rays and 227,827 MedIMG studies. It was created at the investigation level, which is an accumulation of photographs connected with a specific report.

As a result, many chest X-rays with frontal or lateral views can be included in a single scan. The dataset contains a sum of 2,50,100 frontal-view pictures and 1,20,300 lateral-view MedIMG. DICOM images are transformed into JPEGs in this collection. JPEG photos are not only less in size, but they are also easier to understand. The photos were de-identified before being converted to JPEG. After extracting picture pixels from the DICOM input file, the image pixels are normalised to the range [0, 255]. It was examined to determine if pixels are inverted and, if needed, images are inverted to ensure the image's greatest pixel appears as white and the low pixel as black. The contrast of the images was then improved using Contrast Limited Adaptive Histogram Equalisation (CLAHE). Finally, the photos are converted to JPEG format with a 95.78% quality factor and an 8-bit bit depth.

### Computational setup

The NVIDIA DGX-A100 system, which consists of High Bandwidth Memory integrated with eight NVIDIA A100 40 GB Graphics Processing Units (GPUs), was utilised for training the DL models (HBM2). A total of 320 GB GPU memory and the highest level of system power consumption of 6.5kW are contained in this system. The code for our techniques was written using the Pytorch framework and the torch vision package. Table 4 shows a single GPU's consumption of memory for each resolution in gigabytes (GB) during training. As the solution improves, so does the amount of memory used. It depicts the computing capabilities and power required to carry out the experiments. Four A100 GPUs were used for a resolution of 256 × 256 and 512 × 512; however, six A100 GPUs are used simultaneously for 1,024 × 1,024 and 2,048 × 2,048 resolutions to speed up the training. As the image size is doubled, the number of trainable parameters increases, while the number of photos per second drops throughout validation.

**Table 4:** Test setup for hybrid model.

**Image resolution**	**Execution time (s)**	**Memory (GB)**	**Image/s**	**GPU**
256 ×256	30.10	10.39	250	4
512 ×512	112.90	21.39	75	3
1,024 ×1,024	267.18	35.56	25	5
2,048 ×2,048	1,024.48	38.38	7	6

A fascinating result of transfer learning using synthetic data. By changing the rate of training data associated with earlier studies, we test the reduced demand for real data for training the HWNet v2 model. We use the IAM dataset as our testbed again, but we are using a different composition of real data to compare performance with a model that uses all of the training data. Full is represented by 1.0, whereas 0.0 denotes the usage of just synthetic data. It is worth noting that all of these test results are trained on the completely synthetic data set before being fine-tuned with different proportions. As noticed, the performance reduction with less real training data begins slowly and only lowers by 6% when just 10% of genuine data is used. Although this Table 5 shows a lower reliance on real data, a more in-depth investigation (beyond the scope of this project) is required to assess the difference in domain space among synthetic data and handwritten target styles.

**Table 5:** Assessment of word using mAP by hybrid model.

**Training model (%)**	1.0	0.9	0.8	0.7	0.6	0.3	0.0
**Hybrid model**	0.9567	0.9278	0.9187	0.93782	0.8919	0.88192	0.52762

### Compression of embedding representation

**Figure 13:**
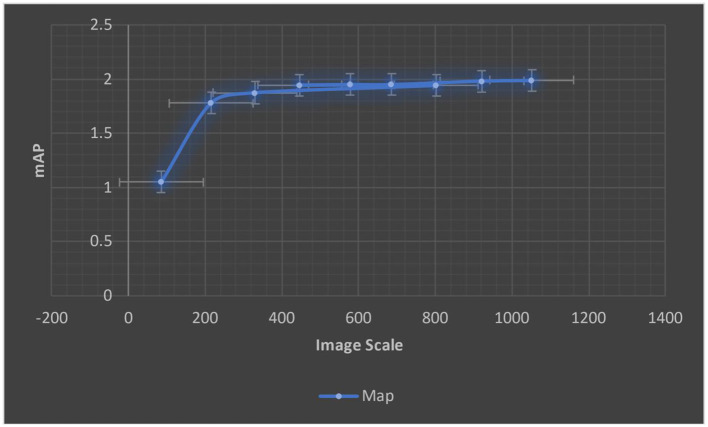
Performance analysis of learning hybrid model using MedIMG.

There is inspiration from our prior studies on integrating the representation into a two-dimensional space, where we discovered surprising similarities between neighbours even under great compression. We now use Principal Component Analysis (PCA), a well-known linear dimensionality reduction technique, to explicitly obtain a lower-dimensional representation of HWNet characteristics. We retrieve the top eigenvectors within representation space from the validation data and use them to project each of the test data. On MedImg datasets, Figure 13 presents the performance difference across variable compression levels ranging from 4 to 1,024. Note that the original HWNet features are 2,048 bytes in size. It is interesting to note that performance in the interval of 32–1,024 dimensions suffers little or no degradation, and we even notice a slight performance boost for 128 dimensions. This shows that the initial HWNet network may capture non-linear data relationships, but the final representing space exclusively contains linear components. On MedIMG with 32 sizes, we achieve an mAP of 0.8942, which is still state-of-the-art when compared to other related approaches shown in Table 6. It is worth noting that the performance attained with only eight dimensions (0.5942) remains superior to several non-DL techniques.

**Table 6:** Analysis of word on MedIMG.

**Model**	**Supervision**	**Dataset 1**	**Dataset 2**	**Dataset 3**
Hybrid	YES	0.9618	0.97182	0.9827

We want to compare the benefits of supervised vs. unsupervised learning, as well as compare performance on such datasets having in-depth features. Table 6 shows the results, which compare HWNet v2 (TPP) to previous techniques. For the English dataset, we apply the pre-trained model by IIIT-HWS with no fine-tuning; however, for the Hindi as well as Telugu datasets, we fine-tune their respective training corpus. The results show that word spotting performance has significantly increased on all of these datasets and that for printed English, HWNet v2 could be used off-the-shelf for various document tasks. The improved results in multi-languages show that architecture could be applied to a wide range of languages with different scripts and language elements. The qualitative outcomes displayed in the last two rows of Figure 13 also demonstrate the level of degrading in the retrieved words, indicating the efficiency of the suggested characteristics (Table 6).

### The outcome of hybrid model (RNN (HWNet v2) + LSTM + GRM)

The Hybrid model has the maximum area under the curve (AUC) for all 14 labels, as shown in Table 7. For each label, an AUC is at least 0.4% greater when compared with the outstanding performance of the single-scale model inclusive of the 1.1% average AUC, which is greater than the model of 1,024 × 1,024. This gap suggests that specialisation happens across scales to any given label, with certain examples best suited to other scales, so that the ensemble can be used to increase accuracy. Table 7 shows that the AUC values acquired for individual models closely resemble the learnt weights. The 1,024 × 1,024 scale, which seems to be the best performing unique model (bold), has been assigned the maximum weight (32.7%) in computing prediction for “Aches, Pain, Fever” with the Hybrid model. Ten of 15 tasks fall under this category.

**Table 7:** AUCs for 15 TRAINED MedIMG.

**ID**	**Finding**	**256 ×256**	**512 ×512**	**1,024 ×1,024**	**2,048 ×2,048**	**Hybrid model**
1	Aches, pain, fever	81.8 ± 0.7	82.7 ± 0.2	81.9 ± 0.4	80.9 ± 0.5	82.4 ± 0.51
2	Addison disease	81.6 ± 0.4	81.67 ± 0.56	81.2 ± 0.5	79.9 ±0.4	82.6 ± 0.41
3	Adult brain tumours	82.1 ± 0.7	82.7 ± 0.2	82.5 ± 0.3	81.0 ± 0.2	83.5 ± 0.4
4	Agoraphobia	88.19 ± 0.3	89.18 ± 0.4	90.20 ± 0.2	89.23 ± 0.3	90.4 ± 0.3
5	Allergy	73.19 ± 0.9	73.8 ± 0.8	73.3 ± 0.8	72.3 ±1.1	74.8 ± 1.5
6	Anaemia	67.7 ± 1.9	67.14 ± 1.4	67.23 ± 1.3	65.6 ± 0.9	68.7 ± 1.7
7	Dandruff vs. dry scalp	73.8 ± 0.9	75.0 ± 0.9	74.5 ± 0.7	73.8 ± 0.5	77.0 ± 0.9
8	Diabetes insipidus	74.9 ± 0.4	76.1 ± 0.2	76.3 ± 0.3	75.4 ± 0.4	77.0 ± 0.4
9	Dialysis	85.3 ± 0.4	85.8 ± 0.4	85.9 ± 0.3	85.3 ± 0.2	86.5 ± 0.15
10	Eczema facts	91.9 ± 0.4	92.3 ± 0.4	92.3 ± 0.3	91.5 ± 0.4	92.8 ± 0.5
11	Calcific bursitis	80.6 ± 0.3	82.5 ± 1.1	82.2 ± 0.7	81.6 ± 0.5	84.0 ± 0.8
12	Cardiac catheterization	71.4 ± 0.6	72.9 ± 0.4	73.1 ± 0.5	71.6 ± 0.6	74.3 ± 0.5
13	Capsule endoscopy	85.6 ±1.1	87.91 ± 0.718	88.4 ± 0.9	88.7 ± 0.4	90.0 ± 0.7
14	Chest X-ray	89.8 ± 0.4	91.8 ± 0.1	92.13 ± 0.3	92.1 ± 0.2	92.7 ± 0.3
15	Lung cancer	80.5 ± 0.3	81.5 ± 0.3	81.25 ± 0.3	80.17 ± 0.12	82.6 ±0.3

This study evaluates model performance using the AUC metric. The area beneath the Receiver Operating Characteristic (ROC) curve (38) is measured by AUC (39). True Positives (TP) and True Negatives (TN) are improved by a model with a higher AUC (40). Table 3 shows the AUC scores for all 15 labels, including the average, for various scales. Because, throughout training, the application of five-fold cross-validation was prevalent. Those AUC scores are the AUC scores average from each of the five folds. Throughout the standard deviations calculated over the five-folds, the AUC scores are displayed. As per our hypothesis, the AUC score for all labels should grow as the input image size increases, i.e., all the bold values should be in 2,048 × 2,048. However, as the table shows, it is not a fact. Some labels meet up to our expectations, while others do not. The AUC score for “Cardiac Catheterization,” for example, drops as image size increases. When compared to 256 × 256 resolution, a few labels performed well with 512x512 resolution, but their AUC scores drop when the resolution is raised to 1,024 × 1,024 resolution.

**Figure 14:**
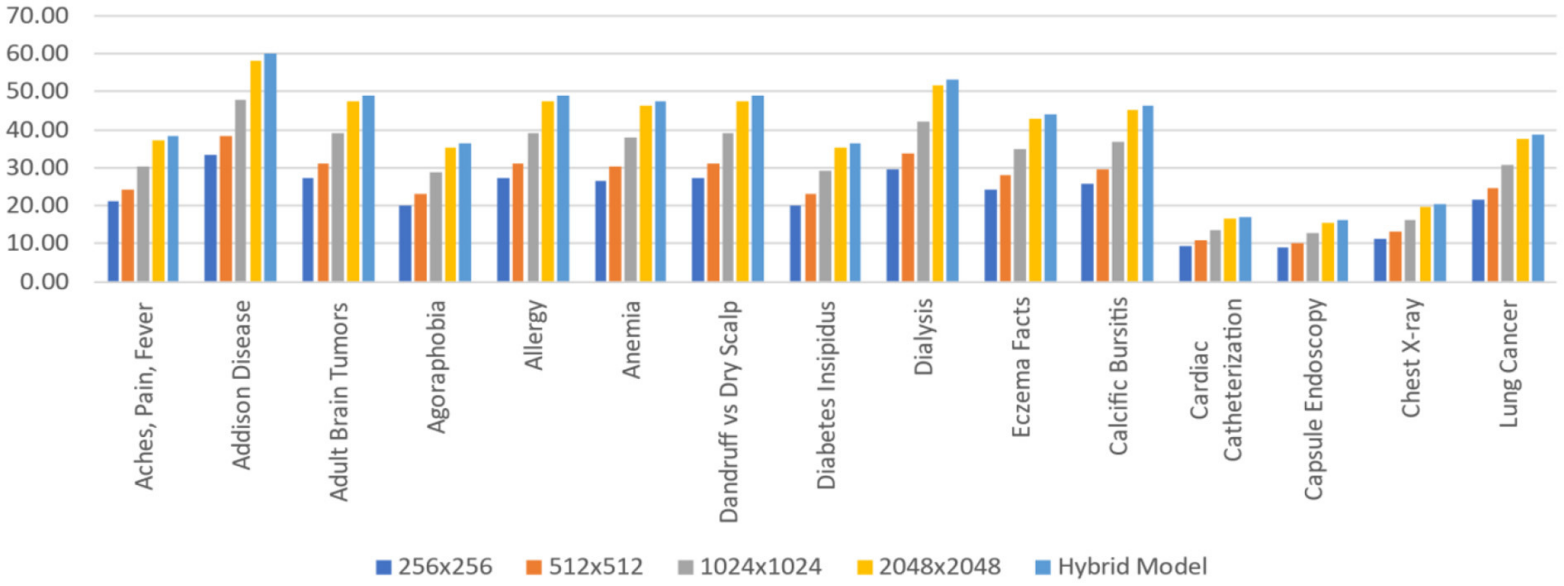
Average stacked scale CNN hybrid model.

For each of the 15 tasks, Figure 14 represents the layered Hybrid Model's learnt weight distribution on four scales. When calculating the predictions for the Hybrid Model, this bar chart displays that resolution is assigned more weights to a specific task. It is worth noting that such weights are given in percentages, and for each task, the weights from all five scales add up to 100%.

### Implementation of MetaMap lite

Table 8 shows the F1scores, Recall, and Precision [42, 43] on every tool to every collection that we are capable of proceeding with it. Because negative detection isn't a natural MetaMapLite feature, and just comparing this one with MetaMapLite implementations of NegEx is to make sure of not losing any performance. We were unable to assess DNorm on two datasets due to difficulty in getting relevant offsets.

**Table 8:** Test report of feature extraction using MetaMapLite.

**MetaMapLite**	**Model**
3 m 31.89 s	Aches, pain, fever
2 m 21.17 s	Addison disease
1 m 34.17 s	Adult brain tumours
2 m 24.28 s	Agoraphobia
3 m 25.38 s	Allergy
2 m 17.38 s	Anaemia
3 m 34.48 s	Dandruff vs. dry scalp
2 m 13.41 s	Diabetes insipidus
4 m 21.38 s	Dialysis
3 m 24.78 s	Eczema facts
2 m 17.89 s	Calcific bursitis
4 m 23.67 s	Cardiac catheterization
2 m 19.89 s	Capsule endoscopy
4 m 18.79 s	Chest X-ray
3 m 23.78 s	Lung cancer

**Figure 15:**
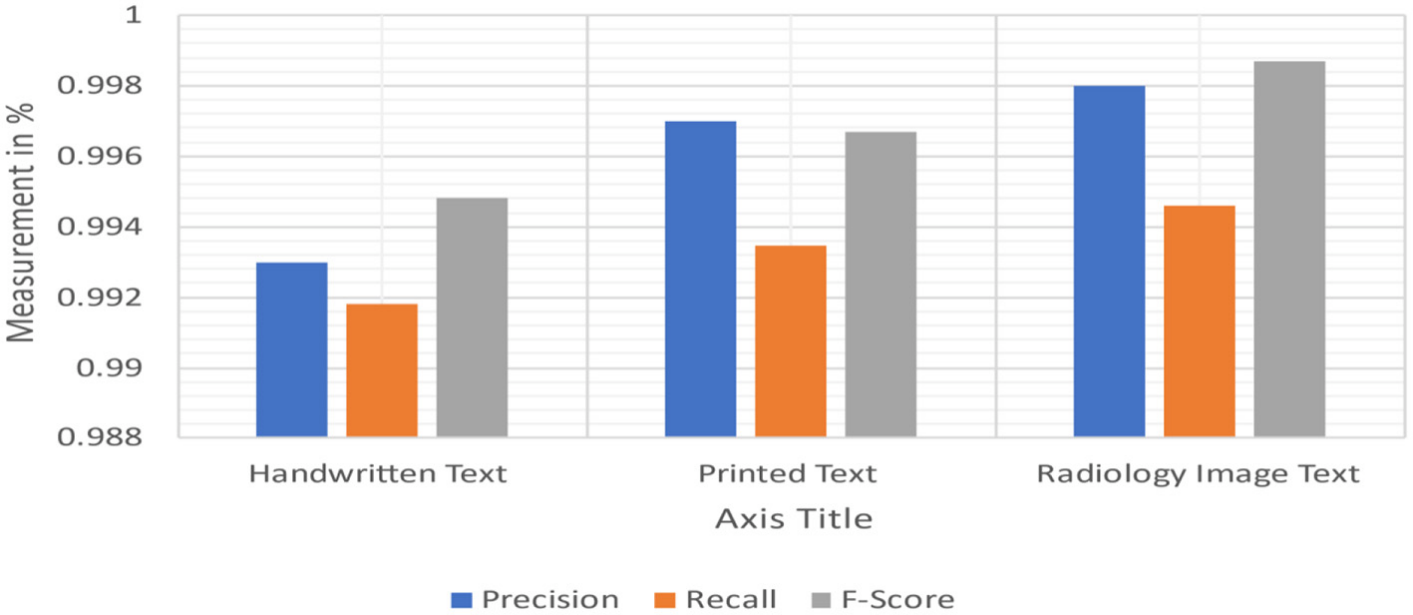
Measuring recall, precision, and F1-scores.

We made considerable effort to install, run, and collect mention-level offsets to third-party programmes. However, with the versions of DNorm available upon this i2b2 2010 and ShARe collections, it cannot obtain mention-level data. It is reasonably sure that the findings we obtained are comparable with those obtained by average end-users of versions of such tools present at the time we conducted our research investigations. On all collections, MetaMapLite received F1scores that were greater than any other tool. The absolute results achieved in our trials may vary from those reported previously, which could be due to differences in tool versions and settings used in our experiments. On request, we may provide all of our tool installations and test collections, which can be made accessible for evaluation scripts. MetaMapLite also takes various restrictions (Figure 15).

It only implements a small portion of the rich MetaMapLite options. MetaMapLite is analysed with the default MetaMap setting, and its performance is equivalent to out-of-the-boxMetaMapLite setups that haven't been tuned for a particular activity.

The processing speed of MetaMapLite is determined by the options selected: the quickest processing is accomplished without POS tagging by a negligible drop in the F-score.

MetaMapLite does not currently have any word-sense disambiguation modules, although it will offer all UMLS senses for a given term. Roughly, mappings that are usually incorrect might be put to a rejection output, similar to MetaMapLite; otherwise, a custom-made data file is handled as a substitute for the tools' regular lexicon records. Finally, the examination has a significant restriction in that it only examines disorders and denials.

## Conclusion

In this paper, Deep Learning is used to extract knowledge from a clever approach to distinguish the doctor's prescription photos and radiology images. The main outcomes are proof of ML methods' ability to discern which elements in short free-text clinical prescriptions are considered inclusion/exclusion criteria, as well as a huge radiology image dataset of images, metadata, radiological results, diagnoses, and anatomic locations. We provide a generic DCNN system for learning document image word image representation. The Hybrid DL Model's core architecture utilises synthetic data to rapidly pre-train multiple data expansion strategies to replicate a typical mechanism at document text formation. Some new visualisation approaches are used to provide other views into the fine-tuning process and to comprehend the differences learned at various levels. On challenging historical texts in both handwritten and radiology modalities, we successfully demonstrate the stability of learned representation in aspects of both performances as well as the dimensionality of the final word. The significance of feature and image resolution in CNN design enabling automated MedIMG categorisation is demonstrated in this study. MetaMapLite, a lightweight software application using MetaMap, is among very widely applied termed NER technique to identify Metathesaurus UMLS ideas as texts on bio-medicine, is described herein our research investigation.

## Data availability statement

The original contributions presented in the study are included in the article/supplementary material, further inquiries can be directed to the corresponding author.

## Author contributions

Conceptualisation and funding acquisition: SB and MA. Investigation and methodology: PD and SB. Project administration: SB. Resources: MA. Writing of the original draft: SS and PD. Writing of the review and editing: SB and SS. Validation and visualisation: PD. Data curation: MA and PD. All authors contributed to the article and approved the submitted version.

## Funding

This work was supported by the Deanship of Scientific Research, Vice Presidency for Graduate Studies and Scientific Research, King Faisal University, Saudi Arabia [Project No. 159].

## Conflict of interest

The authors declare that the research was conducted in the absence of any commercial or financial relationships that could be construed as a potential conflict of interest.

## Publisher's note

All claims expressed in this article are solely those of the authors and do not necessarily represent those of their affiliated organizations, or those of the publisher, the editors and the reviewers. Any product that may be evaluated in this article, or claim that may be made by its manufacturer, is not guaranteed or endorsed by the publisher.
